# Chronic Microvascular Complications in Sulfonylureas-Treated Diabetic Patients: Correlations with Glycemic Control, Risk Factors and Duration of the Disease

**DOI:** 10.3390/clinpract15010007

**Published:** 2024-12-30

**Authors:** Luminita-Georgeta Confederat, Roxana Stefan, Mihaela-Iustina Condurache, Oana-Maria Dragostin

**Affiliations:** 1 Department of Biomedical Sciences, “Grigore T. Popa” University of Medicine and Pharmacy of Iasi, 700115 Iași, Romania; mihaela-iustina.condurache@umfiasi.ro; 2 “Providența” Medical Center, 10 Ștefan Cel Mare Avenue, 700259 Iași, Romania; roxana78ro@yahoo.com; 3 Research Centre in the Medical-Pharmaceutical Field, Faculty of Medicine and Pharmacy, “Dunarea de Jos” University of Galati, 800008 Galati, Romania; oana.dragostin@ugal.ro

**Keywords:** diabetic patients, sulfonylureas, retrospective study, chronic complications, risk factors

## Abstract

**Background**: Diabetes has become one of the most challenging public health problems due to the alarming increase in prevalence and the morbidity and mortality attributed to its acute and chronic complications. **Objective:** This study aimed to investigate the development of chronic microvascular complications in sulfonylureas-treated diabetic patients and their correlations with glycemic control, risk factors and duration of the disease. **Methods**: This study included 200 patients that presented to “Providența” Medical Center, Iași. The information was obtained in a retrospective manner based on the observation sheets of the patients. A database was created, analyzed and statistically processed using the *Microsoft Excel software* (Version 15) and the *chi-square test of independence*. **Results**: The prevalence of diabetic polyneuropathy was 33.5%, while diabetic retinopathy was found in 27% of cases. For diabetic polyneuropathy, the results of the statistical analysis demonstrated a statistically significant dependence of the risk factors hyperlipidemia (significance level = 0.01) and overweight/obesity (significance level = 0.05). For diabetic retinopathy, the results demonstrated a statistically significant dependence of the risk factors hypertension (significance level = 0.05) and hyperlipidemia (significance level = 0.01). **Conclusions**: The present study reveals a strong correlation between the presence of risk factors and the development of microvascular complications of diabetes.

## 1. Introduction

Diabetes has become a major area of interest, both in terms of research activity and in medical practice, as this disease involves considerable medical, social and economic impacts.

Diabetes affects both developed and developing countries. It is one of the most prevalent non-transmissible chronic diseases and the most frequent endocrine disease [1]. According to the International Diabetes Federation’s (IDF) statistics, in 2013, there were 382 million people affected by diabetes, and this number is estimated to rise to 592 million by 2035. In 2015, statistics showed a prevalence of 415 million diabetic patients worldwide, while estimations for 2040 show this figure will increase to 642 million. According to recent IDF statistics, in 2021, there were about 536 million people with diabetes, and the prediction is that 783 million people will have diabetes by 2045, with the highest prevalence in the 75–79 years age group. These data demonstrate an alarming dynamic growth, making diabetes one of the most challenging public health problems [2,3,4]. In addition to this, the global number of deaths attributed to diabetes and its complications has increased considerably, exceeding the cumulated number of deaths caused by HIV infection, tuberculosis and malaria [4].

Known in the literature as “the silent killer”, type 2 diabetes is a silent disease that can evolve asymptomatically for many years. If it remains undiagnosed, untreated or poorly controlled for a long time, it can lead to severe vascular complications. Generally, chronic complications of diabetes occur 15–20 years after diagnosis of the disease. However, there are patients who do not develop vascular complications at all, while some patients already have these complications at the moment of diagnosis [5].

Poorly controlled hyperglycemia leads to multiple vascular complications affecting both small and large blood vessels. Thus, diabetes is a disease of the whole organism, touching, directly or indirectly, all tissues and organs [1]. The most cited chronic vascular complications are diabetic polyneuropathy, retinopathy and nephropathy, with their development being correlated with risk factors such as duration of the disease, poor glycemic control and other co-associated comorbidities [6,7].

The pharmacotherapy of diabetes represents a continuous challenge, both for practitioners and researchers, in terms of understanding the pathophysiological mechanisms involved, developing new therapeutic agents with a more favorable pharmaco-toxicological profile and establishing an individualized treatment regimen with the available drugs that is adequate for each patient’s profile.

Considering the fact that efficient control of blood glucose levels is essential in reducing the development and evolution of diabetes complications, there is a pressing need for the discovery of new effective and safe therapeutic agents [8,9,10].

Antidiabetic sulfonylureas were the first oral hypoglycemic drugs used in the treatment of type 2 diabetes; they represented the first-line medication for patients with mild or moderate diabetes when diet, physical exercise and weight loss could not maintain optimal glycemic control [11,12].

Although the drug of first choice is metformin according to the latest international recommendations, second- and third-generation sulfonylureas remain a feasible therapeutic option, especially in cases of intolerance or contraindications for metformin. Also, sulfonylureas are present in the new guidelines’ recommendations and are prescribed as a single drug or in association with other pharmacological classes [13].

Even though, according to some publications, sulfonylureas should be recommended with caution because of the risk of weight gain, hypoglycemia and poorer outcomes [14], large controlled randomized trials have not shown significant differences in cardiovascular risk, microvascular complications or death between sulfonylureas and other drug classes [15]. This study is focused on analyzing the development of chronic microvascular complications in sulfonylureas-treated diabetic patients and their correlations with glycemic control, behavioral and pathological risk factors and duration of the disease.

## 2. Materials and Methods

This study included 200 patients that presented to “Providența” Medical Center, Iași. The inclusion criteria were as follows: adults diagnosed with type 2 diabetes who had been treated for at least 2 years in outpatient mode with oral hypoglycemic sulfonylureas, both as a single medication and in combination with other oral hypoglycemic drugs, whose evolution could be followed every 3 months within their presentation to the medical center. Patients treated with insulin and patients whose evolution in terms of glycemic control and the presence of diabetes-related complications could not be followed in a sustained manner were excluded from this study. The information was obtained in a retrospective manner by analyzing data included in the observation sheets of the patients. The period of data collection was January 2018 to December 2019. A database was created, analyzed and statistically processed using the *Microsoft Excel software* and the *chi-square test of independence*. This study was approved by the ethical committee of the medical center (approval no. 210/30.01.2018).

In this study, we analyzed the demographical characteristics of the patients; the presence of physiological, pathological and behavioral risk factors for the development of diabetes; duration of the disease; other associated diseases; and the drugs administered. In addition to this, the development of chronic complications and the reported side effects were monitored. This study focused on the development of chronic vascular complications and their correlation with glycemic control, duration of the disease and the presence of risk factors.

## 3. Results and Discussions

The age of the studied patients ranged from 37 to 93 years, with an average of 67.2 ± 9.9 years. The sex distribution was equilibrated (51.5% men and 48.5% women), and most of the patients (62.5%) were living in urban areas. This last aspect could be relevant for the present study, considering the fact that, according to the literature, urbanization is associated with pollution, transition to a Western diet and a sedentary lifestyle, all of which lead to difficulties in the management of diabetes and other cardiovascular risk factors (obesity, dyslipidemia and hypertension) and thus significantly contribute to the development of diabetes complications [16].

Among the patients followed, the duration of the disease (from the time of diagnosis to the time of study) ranged widely, between 2 and 30 years, with the majority of patients being diagnosed 5 to 10 years ago. Related to this, we have to admit that these data are approximates because, according to the literature, type 2 diabetes is a disease that is diagnosed late, with 40% of cases being registered after the development of complications [17].

### 3.1. The Development of Chronic Diabetes Complications

Poorly controlled hyperglycemia can lead to multiple complications, particularly micro- and macrovascular ones. The damage to small blood vessels is the basis for the development of the most well-known serious chronic complications of diabetes: polyneuropathy, retinopathy and nephropathy. The macrovascular complications are related to the atherosclerosis of the blood vessels and are expressed as different clinical types of coronary diseases, including myocardial infarction, heart failure, stroke, peripheral arterial disease and vascular impotence [5,18].

The mechanisms through which vascular damage is caused include glycosylation of the serum and tissue proteins (hemoglobin, plasmatic albumin, collagen, proteins of the crystalline and low-density lipoprotein (LDL) cholesterol); activation of the polyol pathway with accumulation of sorbitol (which, when transformed into fructose, has a rate of glycosylation 7–8 times higher than glucose); production of oxygen reactive species; and activation of protein kinase C, a molecule that is involved in increasing vascular permeability and endothelial dysfunction. In addition to this, the damage of the small and large vessels is amplified by hypertension, dyslipidemia, arterial micro-thrombosis and the pro-inflammatory and pro-thrombotic effect of hyperglycemia [5,13,18].

Consequently, strict glycemic control, early diagnosis of diabetic complications and the management of the risk factors involved could stop or delay the development of diabetic complications or their evolution once they appear.

**Figure 1:**
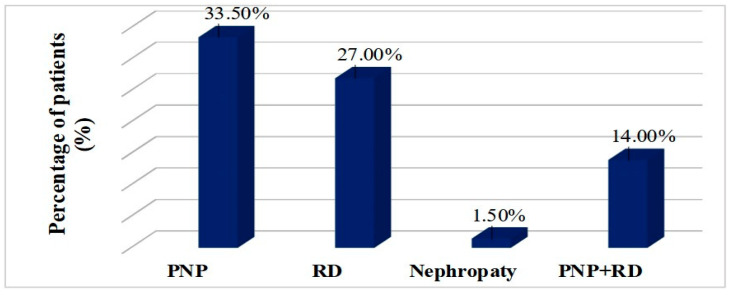
Frequency of microvascular diabetes complications. PNP = diabetic polyneuropathy; RD = diabetic retinopathy.

Within the studied group, the prevalence of microvascular complications is presented in Figure 1. As can be seen, the frequency of microvascular complications, especially diabetic polyneuropathy (PNP) and diabetic retinopathy (RD), was relatively high. Symptomatic PNP was reported in 67 patients (33.5%), described as paresthesia and pain of moderate or severe intensity, especially in the lower limbs. RD was present in 54 patients (27%), of whom only 10 patients were in the non-proliferative stage, with the rest being diagnosed with advanced forms of retinal damage. In addition to this, 28 patients (14%) presented with simultaneous retinopathy and polyneuropathy.

### 3.2. Correlations with Glycemic Control

Efficient long-term glycemic control seems to be the key factor in preventing the development of vascular complications of diabetes. Unfortunately, once they have appeared, these complications cannot be cured by adequate glycemic control, and the main approach is to delay their exacerbation. Taking into consideration these aspects, it is difficult to correlate the development of microvascular complications with the efficiency of glycemic control because the entire medical history of the patients needs to be documented, including the time of onset of the complications.

This study revealed that 21 (31.2%) of the 67 patients that developed diabetic polyneuropathy and 22 (40.7%) of the 54 patients that developed diabetic retinopathy presented with very poor glycemic control, although they had three associated oral antidiabetic drugs in maximal doses; all patients would need to start insulin treatment. Also, 31 (46.2%) patients with diabetic polyneuropathy and 26 (48.1%) patients with diabetic retinopathy presented with moderate to poor glycemic control, characterized by oscillating blood glucose values, due to deviations from the diet and intermittent treatment. However, there were 15 patients with diabetic polyneuropathy and 6 patients with diabetic retinopathy who presented acceptable glycemic control but had several associated pathological and behavioral risk factors. Similar results were reported for patients with diabetic nephropathy, as they presented poor glycemic control despite being treated with three oral antidiabetic agents in maximal doses as well as deviations from diet and interruption of treatment. However, this study has some limitations. Firstly, it is known that non-pharmacological interventions in type 2 diabetes, namely diet and physical activity, are important tools in the management of glycemic control and risk factors. Among the patients studied, information on physical activity was not available from the observation sheets, and, in some cases, there were deviations from the recommended diet; however, this information was obtained in a declarative manner, and it was not available for all the patients in order to be included in the correlations. Secondly, the adherence to treatment was difficult to quantify in this retrospective study, which could generate bias in the interpretation of the results.

### 3.3. Correlations with Duration of the Disease and the Presence of Risk Factors

**Figure 2:**
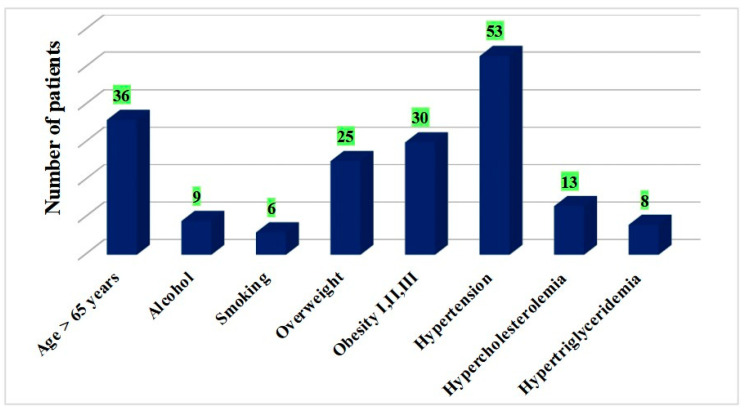
The presence of risk factors and PNP.

In the present study, from the 67 patients who presented with diabetic polyneuropathy as a complication, a significant number presented with a duration of the disease of 5–10 years (36 patients) or 10–20 years (25 patients), with the rest being situated in the time interval of 2–5 years. Concerning physiological and behavioral risk factors, 36 patients were aged over 65 years, while 6 and 9 patients reported smoking and chronic alcohol consumption, respectively. Pathological risk factors (overweight/different degrees of obesity, hypertension and dyslipidemia) were found in the majority of patients with diabetic polyneuropathy (Figure 2).

In order to establish the correlation between the development of diabetic polyneuropathy and the presence of risk factors, the data were statistically analyzed using the *chi-square test of independence (χ^2^).* The results are summarized in Table 1.

Among the patients included in this study, we found a statistically significant dependence between diabetic polyneuropathy and the presence of the risk factors overweight/obesity and dyslipidemia, while the presence of other risk factors, such as age over 65 years, alcohol consumption, smoking and hypertension, did not seem to be statistically significantly related to the development of this microvascular complication. These results are partially in accordance with the literature data. Clinical observations, epidemiologic evidence and animal models of disease have strongly highlighted the association between metabolic syndrome and peripheral polyneuropathy, with the mechanisms being linked to the systemic inflammation induced by obesity and the toxic effects of dyslipidemia [19].

**Figure 3:**
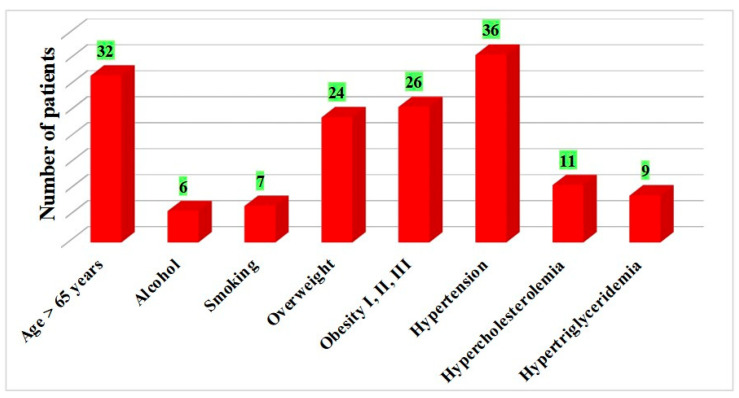
The presence of risk factors and RD.

Concerning the 54 patients that developed diabetic retinopathy and their disease duration, 23 patients were situated in the time interval of 5–10 years, 25 in the time interval of 10–20 years, and the rest in the time interval of 2–5 years. Age over 65 years was reported in 32 patients, while pathological risk factors were found in the majority of patients with diabetic retinopathy (Figure 3).

The correlation between the development of diabetic retinopathy and the presence of risk factors was statistically analyzed using the *chi-square test of independence (χ^2^)*, and the results are presented in Table 2.

Based on the existing literature [20,21], a positive correlation between alcohol consumption and smoking and the development of diabetic microvascular complications was expected. The different findings in this study could be explained by the lower number of patients included and the retrospective design of this study.

**Figure 4:**
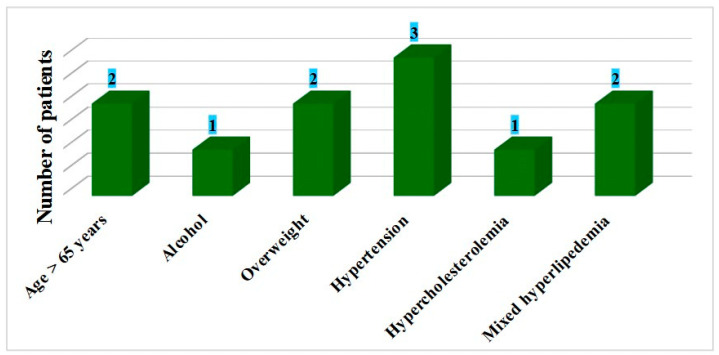
The presence of risk factors and diabetic nephropathy.

Diabetic nephropathy, which is cited in the literature as a rare but severe complication with hyperglycemia and hypertension as the main risk factors, was found in only three patients. In these patients, the duration of diabetes was 8–10 years, and they presented many associated risk factors (age, chronic alcohol consumption, overweight, hypertension and dyslipidemia). It is important to mention that diabetic nephropathy did not appear as an isolated complication, being associated, in all three cases, with polyneuropathy and retinopathy (Figure 4).

In the majority of patients who presented many diabetic complications simultaneously, three or more associated risk factors were reported. However, there were some exceptions. From the 31 patients that concomitantly presented more microvascular complications, two patients diagnosed with diabetes 1 year and 11 years ago, respectively, had evolved polyneuropathy and retinopathy without the evidence of risk factors. Also, two patients with the previously mentioned complications had a long duration of the disease (19 and 25 years, respectively) and presented with only age and overweight as risk factors. This aspect could be explained by poor glycemic control at some point during the disease’s evolution, leading to diabetic complications that could not be treated despite efficient control of all risk factors.

## 4. Conclusions

The present study reveals a strong correlation between the presence of risk factors and the development of microvascular complications. More than half of the patients aged over 65 years, a significant percentage (approximately 50%) of smokers or chronic alcohol consumers, about 60% of overweight/obese patients, about 60% of patients with hypertension and about 40% of patients with hyperlipidemia developed different types of microvascular complications.

The results of the statistical analysis support the fact that the development of microvascular complications is a complex phenomenon, which is conditioned, favored or determined by different risk factors with varying contributions. The results of the statistical analysis using the *chi-square test of independence* demonstrated that, for diabetic polyneuropathy, there was a statistically significant dependence of the pathological risk factors hyperlipidemia (calculated χ^2^ of 17.22 compared to theoretical χ^2^ of 6.63, with a level of significance of 0.01) and overweight/different degrees of obesity (calculated χ^2^ of 5.88 compared to theoretical χ^2^ of 3.84, with a level of significance of 0.05). Regarding diabetic retinopathy, there was a statistically significant dependence of the risk factors hypertension (calculated χ^2^ of 4.45 compared to theoretical χ^2^ of 3.84, with a significance level of 0.05) and hyperlipidemia (calculated χ^2^ of 6.635 compared to theoretical χ^2^ of 6.63, with a significance level of 0.01).

In the evolution of diabetes complications, many factors are involved in different proportions. The implication of the duration of the disease is difficult to appreciate, with these complications generally occurring 15–20 years from the onset of symptomatic hyperglycemia. However, in some cases, these complications might appear earlier. Control of risk factors and blood glucose values is important in delaying complications.

The results of the present study could support some recommendations for clinical practice. Considering the fact that type 2 diabetes is a chronic disease that is naturally progressive, the management of risk factors is crucial for the prevention or delay of the onset of complications. It is well known that type 2 diabetes is frequently associated with obesity and that weight gain is one of the most concerning side effects of sulfonylureas. Considering these aspects and the correlation between obesity and diabetic polyneuropathy, body weight should be monitored, and interventions for weight loss should be implemented in these patients. Additionally, hypertension and dyslipidemia, together with hyperglycemia, are included in the definition of the metabolic syndrome that is frequently present in type 2 diabetic patients; thus, the control of blood pressure values and the lipidic profile should be recommended and monitored at each medical check-up.

This study has some limitations due to its retrospective design and the lack of information on diet, physical activity and adherence to treatment. Additionally, this study was carried out in a single medical center, which limits the generalizability of the findings. Future studies will include multiple centers or regions.

## 5. Future Research

Even if the present study has the above-mentioned limitations, the results open new perspectives for future research. Based on these findings, some interventional trials aimed at controlling obesity, hypertension and dyslipidemia and studying the impact of these interventions on diabetes complications could be conducted.

## Figures and Tables

**Table 1:** Correlations between the presence of risk factors and PNP: statistical analysis.

Theoretical Frequencies (E = Expected Frequencies)			Observed Frequencies (O = Observed Frequencies)			Susceptibility Factors
Total	Neuropathy −	Neuropathy +	Total	Neuropathy −	Neuropathy +	
113	75.145	37.855	113	77	36	Age > 65 years
87	57.855	29.145	87	56	31	Age < 65 years
200	133	67	200	133	67	Total
χ^2^ = Σ(O-E)^2^/E = 0.314234363; degrees of freedom = 1; level of significance = 0.05; probability = 0.95; χ^2^_0.05_ = 3.841; result: independence						Statistical analysis
29	19.285	9.715	29	20	9	Alcohol consumption
171	113.715	57.285	171	113	58	No alcohol consumption
200	133	67	200	133	67	Total
χ^2^ = Σ(O-E)^2^/E = 0.092551086; degrees of freedom = 1; level of significance = 0.05; probability = 0.95; χ^2^_0.05_ = 3.841; result: independence						Statistical analysis
16	10.64	5.36	16	10	6	Smoker
184	122.36	61.64	184	123	61	Nonsmoker
200	133	67	200	133	67	Total
χ^2^ = Σ(O-E)^2^/E = 0.124906686; degrees of freedom = 1; level of significance = 0.05; probability = 0.95; χ^2^_0.05_ = 3.841; result: independence						Statistical analysis
179	119.035	59.965	179	124	55	Overweight/obesity
21	13.965	7.035	21	9	12	Normal BMI
200	133	67	200	133	67	Total
χ^2^ = Σ(O-E)^2^/E = 5.887483775; degrees of freedom = 1; level of significance = 0.05; probability = 0.95; χ^2^_0.05_ = 3.841; result: statistically significant dependence						Statistical analysis
154	102.41	51.59	154	101	53	Hypertension
46	30.59	15.41	46	32	14	No hypertension
200	133	67	200	133	67	Total
χ^2^ = Σ(O-E)^2^/E = 0.251955136; degrees of freedom = 1; level of significance = 0.05; probability = 0.95; χ^2^_0.05_ = 3.841; result: independence						Statistical analysis
104	69.16	34.84	104	83	21	Hyperlipidemia
96	63.84	32.16	96	50	46	No hyperlipidemia
200	133	67	200	133	67	Total
χ^2^ = Σ(O-E)^2^/E = 17.22388635; degrees of freedom = 1; level of significance = 0.01; probability = 0.99; χ^2^_0.01_ = 6.635; result: statistically significant dependence						Statistical analysis

**Table 2:** Correlations between the presence of risk factors and RD: statistical analysis.

Theoretical Frequencies (E = Expected Frequencies)			Observed Frequencies (O = Observed Frequencies)			Susceptibility Factors
Total	Retinopathy −	Retinopathy +	Total	Retinopathy −	Retinopathy +	
113	82.49	30.51	113	81	32	Age > 65 years
87	63.51	23.49	87	65	22	Age < 65 years
200	146	54	200	146	54	Total
χ^2^ = Σ(O-E)^2^/E = 0.22914913; degrees of freedom = 1; level of significance = 0.05; probability = 0.95; χ^2^_0.05_ = 3.841; result: independence						Statistical analysis
29	21.17	7.83	29	23	6	Alcohol consumption
171	124.83	46.17	171	123	48	No alcohol consumption
200	146	54	200	146	54	Total
χ^2^ = Σ(O-E)^2^/E = 0.685253784; degrees of freedom = 1; level of significance = 0.05; probability = 0.95; χ^2^_0.05_ = 3.841; result: independence						Statistical analysis
16	11.68	4.32	16	9	7	Smoker
184	134.32	49.68	184	137	47	Nonsmoker
200	146	54	200	146	54	Total
χ^2^ = Σ(O-E)^2^/E = 2.475569673; degrees of freedom = 1; level of significance = 0.05; probability = 0.95; χ^2^_0.01_ = 3.841; result: independence						Statistical analysis
179	130.67	48.33	179	129	50	Overweight/obesity
21	15.33	5.67	21	17	4	Normal BMI
200	146	54	200	146	54	Total
χ^2^ = Σ(O-E)^2^/E = 0.752842257; degrees of freedom = 1; level of significance = 0.05; probability = 0.95; χ^2^_0.05_ = 3.841; result: independence						Statistical analysis
154	112.42	41.58	154	118	36	Hypertension
46	33.58	12.42	46	28	18	No hypertension
200	146	54	200	146	54	Total
χ^2^ = Σ(O-E)^2^/E = 4.459983138; degrees of freedom = 1; level of significance = 0.05; probability = 0.95; χ^2^_0.05_ = 3.841; result: statistically significant dependence						Statistical analysis
104	75.92	28.08	104	84	20	Hyperlipidemia
96	70.08	25.92	96	62	34	No hyperlipidemia
200	146	54	200	146	54	Total
χ^2^ = Σ(O-E)^2^/E = 6.635314262; degrees of freedom = 1; level of significance = 0.01; probability = 0.99; χ^2^_0.01_ = 6.635; result: statistically significant dependence						Statistical analysis

## Data Availability

The original contributions presented in this study are included in this article; further inquiries can be directed to the corresponding author/s.
